# The Symptom Which Indicates the Remedy

**Published:** 1884-12

**Authors:** T. L. Brown

**Affiliations:** Binghamton, N. Y.


### THE SYMPTOM WHICH INDICATES THE REMEDY.

T. L. Brown, M. D., Binghamton, N. Y.*

Each remedy is individually different in its effect upon the healthy body. That distinctive, tested difference marks its curative relation to the diseased body. But for this individual indication the thorough homœopathic physician cannot be sure he has selected the remedy most likely to cure according to the law of "*Similia.*" The symptom peculiar to only one remedy and not produced by any other, is, when known, the symptom which more correctly indicates that remedy in a case prominently presenting a similar symptom. Practice has proven that when we find in a patient the said objective or subjective symptom, we also find many of the other most common symptoms of the remedy.

For the purpose of inducing physicians to more thoroughly test the individual symptom, and not, as many do, prescribe on general instead of distinctive and demonstrable symptoms, I take this way to call attention to this neglected subject. Life is too sacred to depend, as old school medicine does, upon general, and not upon tested indications for the cure of the sick.

Success is the result of the intelligent use of the most accurately tested similar symptom deciding the best known remedy.

That symptom, like the last added weight that tips the scales, decides the choice of the remedy. Certainly in each prescription is the greatest therapeutic good a physician should aspire to in the use of the remedies. Any suggestion we can add whereby the demonstrable truths peculiar to our school of medicine may be made more intellectually useful is a step in the right direction.

We here offer as many of the individual symptoms without their connecting indications as we have tested in our practice.

We do this to be instrumental in helping others in what we have found useful in the correct selection of remedies.

*Aconite.*—Fear of death, with terrible anguish.

*Æsculus hippocastanum.*—Violent backache in sacro-lumbar region, aggravated by walking or stooping.

*Aloe.*—Feeling of weakness and loss of power of spinctor ani;

---

* Read before Homœopathic Medical Society of New York.

Sense of insecurity in the rectum, as if the stool would escape when passing flatus.

*Alumina.*—Urine can only be passed with the stool, or must stand up to urinate and then sit down to defecate.

*Antimon. crud.*—A thick white coating upon the tongue.

*Apis mel.*—Stinging pains in any part of the body.

*Arnica mont.*—The whole body feels sore and bruised, and is sensitive to touch.

*Arsenicum.*—Violent, unquenchable, burning thirst, with frequent drinking of small quantities of water.

*Asafœtida.*—Flatus passes upward, none downward.

*Asarum eupor.*—Cannot bear the sound of scratching on linen or any similar substance.

*Asclepias tuberosa.*—Feeling as if a stream of fire passed through the abdomen and as if bowels would come out.

*Baptisia tinct.*—Thinks he is double or that his body is broken, and tosses about the bed to get the pieces together.

*Belladonna.*—Moaning during sleep, with half-closed eyes.

*Benzoic acid.*—Dark, strong-smelling urine.

*Bismuthum.*—Thirst; drinks large quantities of water and vomits it immediately.

*Borax.*—Anxious feeling during downward motion or rocking; cannot bear downward motion. (Gels.)

*Bryonia.*—Worse from motion, even of a hand or foot. Better by rest.

*Cactus grand.*—Constriction of the heart as by an iron hand.

*Calcarea carb.*—Sensation as if cold, damp stockings were on the feet.

*Camphor.*—Skin cold as death, but cannot bear to be covered.

*Cantharis.*—Stools white, or pale-reddish mucus stools, like scrapings of the intestines.

*Capsicum.*—Thirst; drinking causing shuddering.

*Causticum.*—Salt-waterbrash.

*Chamomilla.*—Redness on one cheek only.

*Chelidonium maj.*—Constant pain under the inferior angle of the right scapula.

*China.*—Ailments from loss of vital fluids.

*Cina.*—Constantly digging and boring at the nose, with paleness about the mouth.

*Coffea crud.*—Wide-awake condition; mental faculties and senses unusually active; wakefulness at night; will see and hear more readily.

*Colocynth.*—Violent colic, causing the patient to bend double.

*Cocculus.*—Excessive nausea and vomiting when riding in a carriage or when becoming cold.

*Colchicum.*—The smell of fish, eggs, fat meats, or broth causes nausea even to faintness.

*Conium mac.*—Vertigo, particularly when lying down or when turning over in bed.

*Crocus sativa.*—Sensation as if something the size of a fist were rolling around in the abdomen.

*Croton tiglium.*—Sore nipples; when the child draws on the nipple a pain which is excruciating runs through to the corresponding scapula.

*Cuprum met.*—Spasms, with blue face, and thumbs clinched across the palms of the hands.

*Digitalis.*—Weak, slow pulse.

*Dioscorea v.*—Violent twisting colic, occurring in violent regular paroxysms, with remissions.

*Drosera.*—Cough comes in violent paroxysms at intervals of about four hours.

*Dolichos.*—Intense itching of the skin with no appearance of swelling or rash.

*Dulcamara.*—All symptoms aggravated by a cool change of the weather.

*Eupatorium perf.*—Break-bone pains.

*Ferrum met.*—Face flushes easily on the least excitement or exertion.

*Gelseminum.*—Goose flesh over the whole body.

*Graphites.*—Eruption; oozing out of a thick, honey-like fluid, especially behind the ears.

*Hepar sulph.*—Over-sensitiveness to pain.

*Hyoscyamus.*—Immodest; will not be covered; kicks the clothes off.

*Hydrophobin.*—On seeing water or hearing it run, all his symptoms are worse.

*Ignatia amara.*—Suppressed grief. Silent grief, with alternate crying and laughing.

*Iodine.*—Pain in the stomach, relieved by eating.

*Ipecacuanha.*—Constant and continued nausea.

*Jalapa.*—Child "good" all day; screaming, restless, and very troublesome at night.

*Kali bich.*—Stringy ropy mucus. Bladder-like appearance of the uvula, with much swelling, but very little redness.

*Kali carb.*—Wakes about 4 A. M. with cough and stitches in the chest, and stitching pains in the chest.

*Iris versicolor.*—Burning from the mouth to the anus.

*Jaborandi.*—Profuse sweat and profuse salivation.

*Lachesis.*—Always worse after sleep; great mania and loquacity; suspicious even of friends.

*Leptandra.*—Stools like tar.

*Lilium tig.*—A full, distended feeling of all parts of the body.

*Lycopodium.*—All symptoms worse from 4 to 8 P. M. A little food seems to fill the stomach full, and causes fullness and distention of the abdomen.

*Laurocerasus.*—Drink rolls audibly through the œsophagus and intestines.

*Lithium carb.*—Gnawing pains in the stomach; relieved by eating.

*Kreosotum.*—Teeth show dark specks and begin to decay as soon as they appear.

*Magnesia carb.*—Stools with green scum like that of a frog-pond.

*Mercurius corr.*—Tenesmus vesicæ with intense burning in the urethra, and discharge of mucus and blood, with the urine or after it. Urine scanty, hot, bloody, retained or suppressed.

*Mercurius sol.*—Violent tenesmus and continued urging, never-get-done feeling.

*Mezereum.*—Worse in the evening. After suppression of an eruption of thick crusts covering thick pus.

*Muriatic acid.*—Inclination to slide down in the bed.

*Natrum mur.*—General emaciation, most conspicuous about the neck, which is very thin and shrunken.

*Nitric acid.*—Violent cutting and drawing pains in the rectum, continuing for hours.

*Nux vomica.*—Heat with red face and aversion to uncovering.

*Opium.*—Slow full pulse; sleepy but cannot sleep.

*Phosphorus.*—Anus constantly open.

*Phosphoric acid.*—Desire for something refreshing or juicy.

*Plumbum met.*—Sensation of something pulling at the umbilicus with actual retraction of the navel.

*Podophyllum.*—Painless cholera morbus. Stools are profuse and gushing, each seeming to drain the patient dry, but soon he is full again.

*Psorinum.*—Body always has a filthy smell, even after a bath. (Skin dirty, greasy looking.)

*Pulsatilla nig.*—Irresistible desire for fresh air. Worse from warmth or in a warm room.

*Rheum.*—Sour smell of the whole body.

*Rhus tox.*—Tongue dry and rough, with red edges and triangular red tip.

*Rumex crispus.*—Violent dry cough, worse at night; excited by tickling in the larynx, when walking, when talking; by pressure on the larynx or trachea; when lying on the left side.

*Sabadilla.*—Urine thick and turbid, like muddy water.

*Sambucus niger.*—Dry heat of the body with coldness of the feet and hands during sleep; on awakening the face breaks out into profuse sweat, which extends over the body and continues more or less during the waking hours; on going to sleep again the dry heat returns.

*Sarsaparilla.*—Urine deposits white sand. Child screams when urinating. Great emaciation, the skin shriveled and lying in folds.

*Secale corn.*—Aversion to heat, or to being covered.

*Sepia.*—Gone feeling in the stomach, not relieved by eating. Urine turbid, offensive, with reddish or clay-colored sediment, adhering closely to the vessel.

*Silicia.*—Offensive foot-sweat making the feet sore.

*Stannum met.*—The smell of cooking causes vomiting.

*Staphysagria.*—Canine hunger, even when the stomach is full of food.

*Stramonium.*—Loquacious delirium, worse from looking at shining objects; in the dark when alone.

*Sulphur.*—Worse, early in bed. Canine hunger at 10 or 11 A. M. Aversion to washing in cold water.

*Sulphuric acid.*—Sensation of trembling without visible trembling.

*Taraxicum.*—Mapped tongue, with dark red, sensitive places.

*Tartar emet.*—Continuous anxious nausea, straining to vomit with perspiration on the forehead.

*Terebinthina.*—Excessive tympanitis. Violent strangury. Urine cloudy and smoky.

*Thuja.*—Stools forcibly expelled; copious. Gurgling like water from a bung-hole.

*Veratrum album.*—Cold sweat on the forehead.

*Zincum met.*—Feet constantly in motion.

*Zingiber.*—Worse after drinking impure water.

